# Cancer care in the time of COVID-19—a perspective from Pakistan

**DOI:** 10.3332/ecancer.2020.1026

**Published:** 2020-04-20

**Authors:** Aasim Yusuf

**Affiliations:** Shaukat Khanum Memorial Cancer Hospital and Research Centres, Lahore and Peshawar, Pakistan

**Keywords:** cancer, COVID-19, Pakistan, treatment delay

## Abstract

Across much of the world, cancer care has been sidelined to a variable degree by the global effort against the coronavirus pandemic. This paper discusses the impact of coronavirus infection on cancer diagnosis and treatment in two leading cancer centres in Pakistan. It also describes the effect that preparations for the expected surge in cases in Pakistan over the next few weeks have had on cancer care. There is an urgent need to evaluate the effect of delays in diagnosis and treatment on cancer stage and treatment, and to decide how to minimise these during likely future cycles of lockdown over the coming months and years.

With a population of around 220 million, it is estimated that Pakistan has between 170,000 and 200,000 new cancer cases each year [1]. The Shaukat Khanum Memorial Trust operates state-of-the-art comprehensive cancer centres, the Shaukat Khanum Memorial Cancer Hospital and Research Centres (SKMCH&RC), in Lahore and in Peshawar, built and run largely by charitable donations. Each year, approximately 45,000 new patients with cancer come to these hospitals, in the hope of being accepted for treatment. Capacity constraints mean that while we are able to take in close to 10,000 new cancer cases each year, we are forced to refer the remaining patients to other facilities, usually run by the state. 11% of patients seen are children, and patients come not only from all over Pakistan, but also from all provinces of Afghanistan [2].

The Trust operates a unique funding model, supported by charitable donations collected from within Pakistan and from the Pakistani diaspora, supplemented by some clinical income and by proceeds from a nationwide pathology laboratory collection centre network. It treats more than 75% of all patients accepted for cancer treatment completely free of charge, irrespective of race or nationality.

The national health structure is fragile, fragmented and patchy, with islands of excellence in a sea of want. As an indicative example, and with particular relevance to the current situation, the country has approximately 4,000 ventilators for a population of 220 million. The US, also short on supply, has 160,000 for a population of about 328 million.

The first cases of coronavirus surfaced in Pakistan in late February, 2020. Numbers remained low initially, before increasing rapidly as some 3,000 religious pilgrims returned to Pakistan, with many having been infected abroad. The authorities were, and continue to be, heavily criticised for a somewhat chaotic and disjointed initial response, but most of these individuals were quarantined, albeit in sub-optimal conditions. Further increases were seen through March, 2020 as Pakistanis overseas, primarily in heavily infected countries in Europe and in the US, rushed home before travel became impossible or as actual or feared job lay-offs forced them to do so. A recent paper by Bhutta *et al* [3] suggests that evidence of low-level community spread may have started to emerge by early March, 2020.

Patchy social distancing measures have been advised, but inexplicably, large religious gatherings, including regular congregational prayers, have still not been fully proscribed. Educational institutions, offices and many businesses have been closed for more than 4 weeks at the time of writing. Today, most of the country has been in a state of partial lockdown for over 2 weeks, but authorities have been reluctant to impose a complete lockdown for fear of the economic havoc this is likely to wreak on a society where up to 25% of workers are daily-wagers. Recent data from India suggests that such fears are probably well-founded [4]. Balancing the risks of COVID-19 against the risks of real economic deprivation, and starvation, is an unenviable task many governments in low- and middle-income countries will face in the weeks and months ahead.

Cancer care is complex, expensive, often prolonged and difficult for patients and their families at the best of times. Economic uncertainty, restrictions on travel and curtailment of ‘normal’ clinical activity in hospitals as a result of the COVID-19 crisis complicates it further. At SKMCH&RC, Lahore, we have just under 200 inpatient beds and are not, therefore, a large hospital by public-sector standards. It made little sense for us to offer ourselves up as a quarantine centre, or as a ‘general’ hospital to admit patients with relatively mild disease, since there are several 1,000+ bed hospitals in the city that have greater capacity to offer such services. Instead, we felt we ought to play to our strengths and offer higher-end services that are in short supply in Lahore and in Pakistan generally. One of the key areas which is woefully lacking (all across the world) is ICU beds and ventilators. We, therefore, devised a plan to expand our ICU in Lahore from 11 to 15 ventilated beds, and then, using available resources, to 35 ventilated beds. An inpatient floor, comprising 50 beds, has been converted to an ICU, to allow this expansion. We have offered to provide manpower, supplies and space, by conversion of a second similarly-sized unit, to double this number again, to 70 ventilated beds. This latter expansion is contingent on the provincial government giving us equipment (primarily ventilators and cardiac monitors) to operationalise these beds. In order to do all this, we temporarily suspended intake of new cancer patients—most were unable to travel, in any case—and rapidly curtailed cancer follow-up visits. Booked elective surgery was brought forward, with surgeons being asked to complete as many procedures as possible by the end of March, 2020. Emergency surgery, all chemotherapy and radiation treatment continue, for now.

A third inpatient unit, formerly a surgical floor, has been made available as a unit for patients with coronavirus infection who are either not yet sick enough to need ICU care, or for those who have recovered and have been stepped down from ICU care. The Hospital has made a strategic decision that, while our own cancer patients will have first rights to these ICU beds, any excess capacity will be made available to any patient who is likely to benefit, and that all care provided to these non-SKMCH&RC patients will be free, if the patient is unable to pay. We currently have no ventilated coronavirus patients.

Our well-established molecular pathology service meant that we were designated, early on in the pandemic, as one of the three national testing sites for COVID-19, a service we have provided for free as part of our contribution to the national effort against the coronavirus.

Radiology services continue to operate, for inpatients and those in the ICU, as well as for those on active cancer treatment, but only essential imaging studies are being carried out, in order to reduce traffic in the departments and to safeguard the health of staff. All elective imaging and endoscopy, and all screening activity, have been stopped altogether.

There is a nationwide shortage of personal protective equipment (PPE), in particular of N95/Filtering Facepiece Particles (FFP) masks. The well-developed textile industry has stepped up quickly and started to manufacture gowns and other protective clothing, and several local manufacturers are now able to produce perfectly adequate surgical masks although not currently in sufficient numbers to meet expected needs. Efforts to produce N95 masks locally have also now commenced, but there is considerable concern as to whether this will happen quickly enough to be of use in the current pandemic. The National Disaster Management Authority is tasked with purchase, import and distribution of essential equipment, including PPE, but is facing an uphill task in sourcing such supplies in the face of a global shortage and enormous demand, worldwide.

Patient attendants and visitors have been severely restricted—not easy in a culture where it is the norm for several people to come to the hospital with a patient attending a clinic, and even harder for inpatients, where every patient expects to have a relative staying in the room with them. Everyone coming to the hospital, and now also all members of staff, undergoes initial screening for symptoms commonly associated with COVID-19 infection, including fever, cough and breathlessness, as they enter the Hospital campus. All those with such symptoms are referred to a temporary triage and assessment centre, which has been established adjacent to, but outside, the main hospital building. Following further assessment here, all those who meet the case definition undergo testing for coronavirus and are either sent home with instructions to self-isolate until their results are available, or are admitted to hospital for further treatment, as needed. This facility is equipped and staffed so as to ensure that all immediate care needed, including testing facilities and plain radiography, up to and including immediate intubation, are constantly available. The triage centre is open 24 hours a day and is now seeing upwards of 150 patients per day although only a small number have needed admission to the hospital, so far.

This facility has allowed us to segregate patients quickly, so that those who have symptoms suggestive of coronavirus infection, or a history of contact or travel, are routed towards this centre, and can be kept apart from those who clearly have no such history. This is especially relevant to our large population of cancer patients, whom we are trying our best to protect from this infection. We have created separate entrances to the hospital, and separate routes within the buildings, for cancer patients to access areas they need to be in, such as the chemotherapy bays, cancer inpatient units, and so forth. Learning from the Italian experience, it might theoretically be possible, if the number of patients being admitted rises substantially, to designate hospitals, or parts thereof, as being for patients with definite COVID infection (red) versus for patients suspicious for COVID (yellow) versus for definite non-COVID patients (green), thus allowing clear segregation of patients to occur early on in the course of the disease.

After a 4-day gap in which no outpatient clinics were held, we have commenced virtual clinics, with 16 speciality clinics now running each day. Pakistan has a very good mobile-phone network, with estimates suggesting that approximately 90% of Pakistanis live within areas that have cell-phone coverage and more than half of all Pakistanis having access to a cell phone. Pakistan has the highest mobile penetration rate in the South Asian region [5] and so we have had over 95% ‘attendance’ rates, with more than half of these consultations being carried out using WhatsApp video calls. A significant proportion of our patients do not have smartphones, and so we are forced to communicate with them by ‘old-fashioned’ voice calls, using the mobile network. Nevertheless, the response has been overwhelmingly positive, with many patients expressing relief that they have not been abandoned. Where investigations are needed, these are ordered online and a screenshot (or SMS message) is sent to the patient, with instructions to have these carried out at one of our collection centres. Prescriptions for drugs are sent out in a similar manner, following appropriateness review by clinical pharmacists, but patients are being forced to buy their medication themselves, whereas most would ordinarily have received them for free at the Hospital. Courier services are also currently closed, preventing us from sending medication to patients. There is already disquiet amongst treating clinicians as to whether our indigent patients will be able to purchase their medication in the current climate, not only for financial reasons, but also because of the expected disruption of drug supply chains.

We have not seen enough cancer patients infected with coronavirus at this stage to be able to see any unusual patterns of the disease specific to cancer patients, but the coronavirus pandemic has certainly had great impact on the treatment we have been able to provide for cancer. In some cases, treatments recommended have had to be changed to reflect the current reality of what is possible and available. Thus, a number of patients with hepatocellular cancer considered suitable for ablative therapy or for chemoembolisation have instead been asked to start oral sorafenib, a much more expensive proposition, but one which does not require immediate visits to the hospital. Similarly, those completing neo-adjuvant chemotherapy are having to wait longer than normal for planned surgery, and may need to have additional cycles of chemotherapy to tide them over the increased waiting time. Individual chemotherapy cycles have been spaced out, and radiation treatments are being given in a more compact manner, with fewer fractions, where possible.

In a typical month, we would normally accept around 850 new cancer patients for treatment. Since the end of March, 2020, no new cancer patients have been accepted for treatment, the number of new patients who have commenced chemotherapy has gone down by two-thirds and the number of patients commencing radiation treatment has halved. While we would normally perform approximately 800 elective surgical procedures for cancer each month, since the lockdown 2 weeks ago, less than 20 emergent surgical procedures have so far been performed. We are concerned that the number of patients presenting with a need for emergency surgery will increase over the next few weeks. Delays in acceptance of new patients and in treatment of patients already in the system are likely to have highly detrimental effects on cancer stage at presentation, on survival and on cure rates, nationally and globally.

Daily counselling sessions have been organised for healthcare and support staff fearful of the risks of working in the current situation. These have been well attended, and well received. Similar sessions have also now been set up for patients in need of psychologic or psychiatric support, using telemedicine facilities.

Given the virtually complete shutdown of routine clinical activity in the country, the Hospital has seen a significant drop in its clinical income, usually largely derived from diagnostic services and the pathology collection centre business described earlier. There is also concern as to the quantum of charitable donations we will collect over the coming year, given the expected massive downturn in the domestic economy and the knock-on effects of the predicted global recession. We have reduced our payroll costs by over 10%, by reducing salary for all staff, ranging from a 5% reduction for the lowest paid to 25% for the highest pay-bracket. Cancer professionals are exceptionally hard to recruit, train and retain in resource-constrained environments, and we are determined not to lay off staff. A number of the highest-paid staff in the hospital have agreed to forego their salaries altogether, for up to 6 months, if further belt-tightening becomes necessary.

As stated above, the Hospital has always been able to accept only a proportion of those who seek cancer care. This is done based on a variety of factors, including age, likelihood of achieving a complete cure, availability of appropriate drugs, personnel, equipment and so forth. While we would like to accept all patients who wish to be treated, we have been forced, for the last 25 years, to make tough decisions on who to accept, and to develop protocols for such rationing of care. This experience has helped us to develop decision-rules for allocation of ICU beds in the current crisis, something that institutions all over the world are now having to do.

We are naturally anxious to ensure that no patient slips through the cracks as a result of the current disruption of service and so oncology fellows have been tasked to maintain a registry of all patients whose treatment or follow up has been impacted by the coronavirus pandemic, or by the measures we have taken to counter this. At the time of writing, Pakistan has over 4,000 confirmed patients with coronavirus infection, 25 of whom are critically ill and ventilated, and 58 patients have succumbed to their disease. These numbers are still relatively low, but testing is very limited, and we are now waiting for the expected peak in late April, 2020.

There is clearly a need to start to model the downstream effects of delay in therapy for cancer patients, especially since it is likely that the coronavirus epidemic will have more than one peak, and governments everywhere are increasingly talking about not one, but a series of lockdowns. Delays to cancer screening programmes also have the potential to impact cancer diagnosis, stage and survival. As cancer professionals, we need to be able to use such data to advocate more forcefully for our patients in future and to allow us to argue more effectively for an equitable share of resources for cancer patients, so as to allow a return to full clinical function as soon as possible.

## Conclusion

While we are acutely conscious of our responsibility to our cancer patients, like healthcare professionals everywhere we are being forced to ration and prioritise our scarce healthcare resources. There is increasing concern amongst cancer professionals all over the world that the longer-term effects of the strategy of lockdowns and varying degrees of closure of ‘routine’ clinical activity, currently in force across much of the world, have not been fully thought through. While this strategy may prove effective against the coronavirus, there are serious doubts as to whether its effects on other diseases, and the implications of delay in treatment of other conditions, have been seriously considered. Any planning of an exit strategy from lockdown and social distancing must take into account the needs of cancer patients and of others with serious conditions who cannot wait, seemingly interminably, for treatment.

## Conflicts of interest

The author has no disclosures.

## Funding statement

No funding was received for this work.
